# Endothelial Nitric Oxide Synthase Gene Polymorphism (G894T) and Diabetes Mellitus (Type II) among South Indians

**DOI:** 10.1155/2011/462607

**Published:** 2011-11-09

**Authors:** T. Angeline, H. R. Krithiga, W. Isabel, A. J. Asirvatham, A. Poornima

**Affiliations:** ^1^PG & Research Deparment of Zoology and Biotechnology, Lady Doak College, Madurai 625 002, Tamil Nadu, India; ^2^Arthur Asirvatham Hospital, Kuruvikaransalai, Madurai 625 020, Tamil Nadu, India

## Abstract

The objective of the study is to find out whether the endothelial nitric oxide synthase (eNOS) G894T single-nucleotide polymorphism is associated with type 2 diabetes mellitus in South Indian (Tamil) population. A total number of 260 subjects comprising 100 type 2 diabetic mellitus patients and 160 healthy individuals with no documented history of diabetes were included for the study. DNA was isolated, and eNOS G894T genotyping was performed using the polymerase chain reaction followed by restriction enzyme analysis using *Ban II*. The genotype distribution in patients and controls were compatible with the Hardy-Weinberg expectations (*P* > 0.05). Odds ratio indicates that the occurrence of mutant genotype (GT/TT) was 7.2 times (95% CI = 4.09–12.71) more frequent in the cases than in controls. Thus, the present study demonstrates that there is an association of endothelial nitric oxide synthase gene (G894T) polymorphism with diabetes mellitus among South Indians.

## 1. Introduction

Prevalence of diabetes is increasing globally, and the worldwide prevalence of diabetes for all age groups was estimated to be 2.8% in 2000 and is predicted to be 4.4% in 2030 by the World Health Organisation (WHO) [1]. The total number of people with diabetes is projected to rise from 171 million in 2000 to 366 million in 2030 [1]. India, China, and the United states are among the top three countries estimated to have the highest number of people with diabetes [2]. The problems associated with this disease are obesity, hypertension, cardiac disease, diabetic retinopathy, renal failure, diabetic neuropathy, albuminuria, and other vascular diseases [3]. 

It has been suggested that diabetes mellitus is a pathological condition where a decreased nitric oxide may present. Nitric oxide (NO) is a substance which is capable of keeping vascular tone, coagulation, and inflammation well balanced. Nitric oxide is synthesized by endothelial cells from L-arginine and molecular oxygen by endothelial nitric oxide synthase. Decreased NO activity can be caused by impaired production of NO, due to uncoupling of receptor-mediated signal transduction, a deficiency of the NO synthase (NOS) substrate l-arginine, or a decreased availability of one or more cofactors essential for optimal functioning of NOS [4].

Apart from environmental factors contributing to type 2 diabetes, such as obesity, smoking, sedentary lifestyle, and certain drugs, genetic factors also play a role in the onset of this disease. Among the genes thus investigated, the endothelial nitric oxide synthase (eNOS) gene has drawn considerable attention because of its substantial contributions to vascular diseases. 

A single-nucleotide polymorphism (G894T) in the gene-encoding eNOS exon 7 has been discovered [5]. It results when guanine residue at position 894 in exon 7 of eNOS gene is replaced by thymine residue (Glutamate—GAG to Aspartate—GAT) at the codon 298 [5, 6]. The prevalence of the polymorphism varies in different population, which may be reflective of different ethnicities. In recent years, eNOS gene polymorphism have also gained enormous attention due to their association with diabetes mellitus regulation.

A previous study has investigated the prevalence of this polymorphism among South Indians [7]. So far, no study has been carried out to elucidate the role of this polymorphism in South Indian patients with type 2 Diabetes mellitus. Therefore, the present case-control study has been extended to find out whether there is any association of this eNOS gene polymorphism with type 2 diabetes mellitus. 

## 2. Methodology

### 2.1. Selection of Subjects

A total of 260 unrelated south Indian (Tamil) males of age 40–55 (50 ± 6.4 yrs), with no history of cardiovascular disease, dyslipidemia, hypertension, renal failures, and so forth, who belonged to south Indian Tamil ethnicity [7] were included for the study. Out of the total 260 individuals, 100 of them were type 2 diabetic patients and 160 of them were nondiabetic controls. Ethical clearance was obtained. The informed consent was obtained from the study subjects. The Tamils represented in this study are the people who not only live in Tamil Nadu, but also belong to ethnic group from Tamil Nadu. The identity has historically been primarily linguistic, with Tamils belonging to those whose first language is Tamil. The patients were characterized as diabetes mellitus based on the fasting blood glucose concentration. Diabetes mellitus was considered to be present if the fasting glucose concentration is greater than 126 mg/mL. Individuals with normal blood glucose concentration (<126 mg/mL) were considered as controls. People with history of cardiovascular disease, dyslipidemia, hypertension, renal failures, and so forth and those who do not belonged to south Indian Tamil ethnicity were excluded from the study population.

### 2.2. DNA Extraction

Genomic DNA was isolated from the frozen blood by salting out procedure. For DNA isolation, 300 *μ*L of the blood was used, and the isolated DNA was stored at −20°C. The isolated DNA was confirmed by 0.7% Agarose (HI-Media, Mumbai) gel electrophoresis and quantified by UV spectrophotometry (Hitachi, Japan).

### 2.3. PCR Analysis of G894T SNP [7]

PCR analysis was carried out as previously described [7] using eppendorf master cycler (Germany). Genomic DNA (~50 ng) was incubated in a total reaction volume of 50 *μ*L containing equal concentration of the forward primer 5′ TCC CTG AGG GCA TGA GGC T-3′ (70 picomoles) and reverse primer 5′ TGA GGG TCA CAC AGG TTC CT-3′ (Gene script corp., USA), 200 *μ*M deoxynucleotide triphosphate, 10X PCR buffer pH-8.3 containing 15 mM Mgcl2 and 1.5 units of Taq DNA polymerase (New England Biolabs, Beverly, Mass, USA).

DNA was initially denatured at 95°C for 5 min prior to amplification. PCR amplification was accomplished using 30 cycles, consisting of 2 min denaturation at 95°C, 45 sec annealing at 62°C, and 1 min extension at 72°C and the final extension included a 1 min extension at 72°C.

### 2.4. Restriction Enzyme Analysis

Restriction digestion was performed in a total volume of 10 *μ*L amplicons, 1 *μ*L NE buffer (50 mM potassium acetate, 20 mM tris-acetate, 10 Mm magnesium acetate, 1 mM dithio threitol pH-7.9 at 25°C) and  8  units of *Ban II* enzyme (New England Biolabs, Beverely, Mass, USA). Samples were then incubated for 5 hrs at 37°C and the digested PCR products were separated by 2.5% agarose gel electrophoresis.

### 2.5. Confirmation of PCR by Direct DNA Sequencing

Selected PCR amplified fragments were completely sequenced both strands in an automated ABI 3100 Genetic Analyser (Bangalore GeNei sequencing services, India).

### 2.6. Statistical Analysis

Categorical data were analyzed using absolute counts and proportions. The 2 × 2 contingency tables were constructed to compare the patients and controls using an odds ratio. The 95% confidence intervals (95% CI) were also calculated. The allele frequencies were calculated by allele counting. Pearson Chi-square (*χ* ^2^) test was performed to find the statistical significance between the observed genotypes and expected genotypes of the selected population.

## 3. Results and Discussion

The “T” allele frequency of the controls obtained in this study (0.15) is comparable to the “T” allele frequency observed in the previous study [7], which was conducted among the same South Indian (Tamil) population. Chi-square analysis at 5% level of significance showed that the study population lies in the Hardy-Weinberg equilibrium (*P* > 0.05).

In the present study, the occurrence of mutant genotype (eNOS-894 GT/TT) was found to be high (75%) in type 2 diabetic patients when compared with the controls (29.37%) (Table 1). A similar study conducted by Monti [8] described a significant association between endothelial nitric oxide synthase (eNOS) gene polymorphism and type 2 diabetes, suggesting a new genetic susceptibility factor for hyperinsulinemia, insulin resistance, and type 2 diabetes mellitus. It has been shown that there is a significant association of eNOS-G894T single-nucleotide polymorphism with end-stage renal disease [9]. A five-year prospective study conducted among Chinese subjects reported that the eNOS G894T polymorphism appears to be predictive of persistent hyperglycemia in individuals with impaired glucose tolerance [10].

A previous study conducted among healthy South Indians reported the absence of homozygous mutant genotype (eNOS-894 TT) [7]. The results of the present study also indicate the complete absence of homozygous mutant genotype (eNOS-894 TT) in the control group among South Indians, whereas another study conducted among North Indians reported the presence of homozygous mutant genotype (eNOS-894 TT genotype) among healthy individuals [11]. Such differences might be attributed to different background or ethnicity that exist between North Indian and South Indian ancestry. However, in the present study, mutant genotype was observed in individuals with diabetes mellitus. 

A study conducted among Asian Indians by Ahluwalia [12] suggested that that the eNOS gene locus is associated with diabetic nephropathy and the functional polymorphisms (−786T > C & 894G > T) might lead to a decreased expression of eNOS gene. It has been reported that the “T” allele is associated with the pathogenesis of diabetic nephropathy in patients with diabetes mellitus [13]. A study conducted among the white Australian population had the increased frequencies of the missense Glu298Asp variant in the eNOS gene, but these were associated with neither the occurrence nor the severity of coronary artery disease [14].

A meta-analysis was conducted among 28 association studies focusing on the three polymorphisms in the NOS3 gene (G894T (Glu289Asp), 4b/a, and T-786C) and the risk of diabetic nephropathy [15]. It was found out that G894T was negatively associated with diabetic nephropathy in Caucasian populations of European origin (OR = 0.896, 0.817–0.983, 95% CI), but was positively associated with DN in East Asian (OR = 2.02, 1.20–3.42, 95% CI) and other populations

In the present study, individuals with the mutant TT genotype has a 7-fold increased risk of developing diabetes mellitus. Mutant “T” allele frequency was also found to be high (0.48) in patients with type 2 diabetes mellitus than the controls (0.15). Thus, heterozygosity/homozygosity (GT/TT) for the G894T eNOS polymorphism has been associated with diabetes mellitus. This may be attributed to the decreased nitric oxide synthase activity in individuals with mutant “T” allele, which in turn may lead to decreased synthesis of nitric oxide. However, there is another study conducted among the same south Indian population associating the eNOS G894T polymorphism and its relationship with coronary artery disease [16]. The “T” allele frequency observed in that study (0.12) is comparable to that which is observed in the present study (0.15). The absence of TT genotype in the control group could be due to its lower frequent in controls, compared to the patient group, and so, control subjects with TT genotype could be found if the sample size was larger.

The GAG to GAT substitution in exon 7 (G894T) determines the conservative replacement of glutamate with aspartate (Glu298Asp), which might cause a tight turn of the *α*-helix and, therefore, an increased susceptibility to degradation [17]. The eNOS G894T variant generates protein products with different susceptibility to cleavage that is, aminoterminal 35-kDa and carboxy terminal 100 kDa fragments [18]. This suggests that even a single aminoacid substitution could have a functional effect on the function of the eNOS protein. Moreover, it has also been demonstrated that this polymorphism involves protein-protein interactions with chaperon proteins that control subcellular trafficking of the enzyme as well as with proteins involved in the degradative processing [8]. 

While homozygous mutant “TT” frequency was found to be high (15%) among Caucasians [19], such condition was found to be very rare in Asian population [20]. Though the South Indians are Asians, the type 2 diabetes mellitus patients among South Indians had “T” allele frequency higher than that of the controls insisting the association of mutant “T” allele in type 2 diabetes mellitus.

The role of nitric oxide as a modulator of physiological insulin secretion has been extensively evaluated [21]. Nitric oxide has the ability to modulate peripheral and hepatic glucose metabolism and insulin secretion [22]. It has been described that eNOS activation, specifically localized to skeletal muscle mitochondria, increases muscle blood flow, with increased delivery of insulin's major substrate, glucose, to the muscle cell. Thus, genetic defect in the eNOS might be a new player in the evolution of hyperinsulinemia and insulin resistance as previously suggested [8].

The frequency of the endothelial nitric oxide synthase eNOS gene single-nucleotide polymorphisms (SNPs) varies among ethnic population [23]. The occurrence of different genotype in different population may depend on race and ethnic background of the population. To the best of our knowledge, this is the first study, to relate endothelial nitric oxide synthase eNOS G894T single-nucleotide polymorphism with type 2 diabetes mellitus patients among South Indians.

To conclude, the present study demonstrates that there is association of endothelial nitric oxide synthase gene (G894T) polymorphism with diabetes mellitus among South Indians. This has to be further substantiated by analyzing large number of samples and also evaluating the level of nitric oxide in patients with G894T variant. Further studies including other eNOS polymorphisms (intron 4b/4a, T786C, repeats in intron 13,1998C/G) and to the related biochemical parameters including nitric oxide estimation would better explain whether there is actual decrease of nitric oxide level in patients with type 2 diabetes mellitus and whether by increasing the NOS activity the risk could be reduced for developing diabetes mellitus. 

## Figures and Tables

**Table 1:** Genotype frequency of the eNOS G894T polymorphism in type 2 diabetes mellitus patients, controls, and total study subjects.

eNOS G894T genotype/allele frequency	Genotype frequency *n *(%)			OR (95% CI)
	Total subjects *n* = 260	Patients *n* = 100	Controls *n* = 160	
GenotypeGG (wild type)	138 (53.07)	25 (25)	113 (71.62 )	7.213 (4.094–12.706)
GT (heterozygous)	102(39.23)	55 (55)	47 (29.37)	5.289 (2.954–9.470)
TT (mutant)	20 (7.69)	20 (20)	—	—
Allele G	0.73	0.52	0.85	6.139 (3.123–12.053)
T	0.27	0.48	0.15	
